# Skater's Cramp: A Possible Task‐Specific Dystonia in Dutch Ice Skaters

**DOI:** 10.1002/mdc3.12799

**Published:** 2019-08-14

**Authors:** Beorn Nijenhuis, Aron H.P. Schalkwijk, Sharon Hendriks, Rodi Zutt, Egbert Otten, Marina A.J. Tijssen

**Affiliations:** ^1^ Department of Neurology University Medical Center Groningen, University Groningen Groningen the Netherlands; ^2^ Department of Neurology Haga Teaching Hospital the Hague the Netherlands; ^3^ Department of Movement Sciences University Groningen Groningen Netherlands

**Keywords:** skater's cramp, task‐specific dystonia, skating

## Abstract

**Background:**

Skater's cramp is an involuntary lower leg movement in skilled speed skaters. We aim to evaluate whether skater's cramp is compatible with task‐specific dystonia.

**Methods:**

A case‐control study tested 5 speed skaters exhibiting symptoms of skater's cramp and 5 controls. Affected skaters completed a standardized questionnaire and neurological examination. Video analyses included skating normally, intensely, and with extra mass around the skater's ankles. An Inertial Motion Capturing (IMC) device mounted on both skates provided angular velocity data for both feet.

**Results:**

Median time of onset of skater's cramp occurred after 12 (range 3–22) years of speed skating. Skater's cramp appeared as task specific; its onset was sudden and correlated to stress and aberrant proprioception. Symptoms presented acutely and consistently during skating, unilaterally in 4 and bilaterally in 1 skater. Visually, skater's cramp was an active, patterned, and person‐specific jerking of a skater's foot, either exo‐ or endorotationally. It presented asymmetrically, repeating persistently as the foot neared the end of the swing phase. The skater's affected leg had a longer swing phase (median, 1.37 [interquartile range {IQR}, 0.35]/1.18 [IQR, 0.24] seconds; *P* < 0.01), a shorter glide phase (median, 1.09 [IQR, 0.25]/1.26 (IQR, 0.29) seconds; *P* < 0.01), and higher angular velocity during the jerking motion. Symptoms remained constant irrespective of speed or extra mass around the ankle (*P* > 0.05). No significant differences between legs were detected in the control group.

**Conclusions:**

Observed clinical, visual, and kinematic data could be an early and tentative indication of task‐specific dystonia.

View Supplementary Video 1

Skater's cramp is a dreaded condition among speed skaters and inline skaters that diminishes their control and stability. Known in the Netherlands as zwabbervoet,^1^, ^2^ skater's cramp has affected various famous Dutch speed skaters, spelling the end of their professional careers. The condition is described as a feeling of lessened control in the affected leg/foot, that only occurs during speed or inline skating.^1^ It visually presents as an aberrant twitch‐like spasm that occurs while a skater's skate is suspended in the air, after it has been retracted from a completed stroke, and before it is placed on the ice to glide again (swing phase). Although the prevalence and pathophysiology is still unknown, suggestions have included flawed skating technique (an adaptation to compensate for a pre‐existing technical inadequacy), sport‐specific compartment syndrome, or peripheral neurogenic damage.^1^ Fascinatingly, little scientific evidence exists to support these claims; therefore, some researchers have recently suggested task‐specific dystonia (TSD) as a possible alternative explanation.^2^

Skater's cramp seems to match well with the principal criteria for TSD, namely, task specificity. In musicians or surgeons for example, TSD arises partly because of maladaptive changes in the sensory‐motor system, caused by practicing a skilled motor ability too much.^3^ It is likely that, because it is partly caused by adaptations to overpracticing, the dystonia often only presents during the execution of the practiced movement and nowhere else. Cases of skater's cramp seem to show a similar pattern, given that a skater's motor abilities remain otherwise unaffected off the ice track. Even more specifically, TSD often only presents in subphases of a broader skilled movement; in musicians, for example, symptoms of TSD are often so specific as to affect one finger during one select movement.^4^ Similarly, initial observations indicate that skater's cramp is also extremely localized around the “swing phase.” In light of this, we chose to test the hypothesis that skater's cramp is a form of TSD.

To investigate this hypothesis, we looked at six major criteria for diagnosis. First, we investigated whether skater's cramp was task specific to speed skating, had a sudden onset, and was possibly related to stress. We further investigated visual features, whether the dystonia was active, patterned, and person specific. Because the left and right leg mirror each other bilaterally during the straight away portion of the skating movement, we performed a visual analysis and additional kinematic movement comparison of affected and unaffected legs. We further compared these movements with healthy skaters. We hypothesized that if the above profiled features accurately described the affected skaters, it would constitute tentative evidence that skater's cramp may be a form of TSD.

## Patients and Methods

A case‐control study comprising 5 speed skaters with skater's cramp and 5 experienced unaffected speed skaters was performed between June and December 2014. Participants were recruited through an open request in the speed skating community. The study was performed on the 400‐m skating track of the Thialf Stadium, Heerenveen and on the 400‐m skating track of Kardinge, Groningen, the Netherlands. The study was coordinated from the University Medical Hospital in Groningen, the Netherlands (UMCG). The Medical Ethical committee of the UMCG approved the study (M13.147574).

### Population

All participants were at least 18 years of age at examination; no further selection on age was considered, given that the subjects were matched according to proficiency level. It was a prerequisite that the skaters were highly experienced and practicing more than twice a week in‐season for at least 5 years without any previous dystonic complications. For the affected speed skaters, skater's cramp had to be present for at least 6 months. Exclusion criteria included other known neurological disorders. Informed consent was obtained from every individual.

All participants answered standardized questionnaires, including demographic and clinical features with detailed questions regarding skater's cramp, age of onset, duration of skating career, rate of onset, provoking factors at onset and description of the symptoms, plus luxating and contributing factors. In addition, all participants underwent a neurological examination conducted by a neurologist specialized in movement disorders (M.T. and R.Z.).

### Standardized Exercise Design

The skaters performed two laps at normal speed, followed by a lap steadily building speed (“steigerung”). This routine was followed by three laps at normal speed with weights on both ankles (0.75 kg each), increasing the mass and the moment of inertia of the foot/skate system.

### Video Recording

Video was captured of skaters, filming them from behind, as they skated away from a stationary camera located at the end of the corner, positioned to record them exiting the corner and skating down the straight section of the 400‐m ice track. In addition, a speed skater (S.H.) pursued the participants with a helmet‐mounted GoPro camera (GoPro Hero 3, 720 p, 47.95 fps; GoPro, San Mateo, CA). Video recordings were saved for extensive visual analysis.

### Motion Capturing

Accelerometer data were recorded using a portable inertial motion capturing system called Motion and Muscle Ambulatory Activity System. The sensor module consisted of five wired inertial motion unit (IMU) sensors, each individually capable of registering three‐dimensional movement variables at 100 Hz (rate of turn [rad/s], acceleration [m/s^2^], and magnetic field strength [mgauss]). The IMU sensors were positioned at the tip of each shoe using a biaxial (X, Y) adjustable platform. With the help of an angle protractor, a baseline yaw and pitch angle close to zero was obtained. Additional sensors were placed halfway up each upper leg and one on the lower back.

### Movement Variables

**Figure 1:**
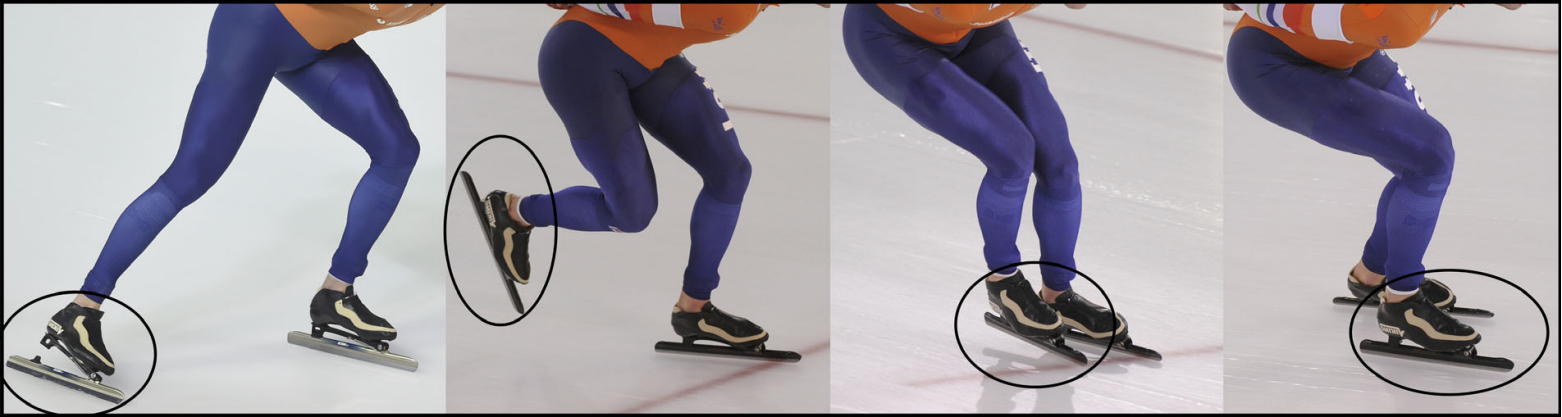
One full gait cycle for a speed skater consists of a push, swing, skate placement, and glide phase. Skate placement is the precarious moment where skaters must shift their center of gravity over the alternate skate while placing it on its outside edge.

Measurements consisted exclusively of skating strides on the straight section of the 400‐m ice track. From the routines at normal speed, total stride time (seconds) and length of each gait cycle phase (seconds) were obtained from the inertial data for all participants. The skating cycle was split into three distinct movement phases for analysis (see Fig. 1): (1) the swing phase, that started after push off and continued until the foot neared the ice (2) the glide phase, which was initiated by skate placement on the ice when weight was shifted on it, after which the skater glided forward, and (3) the push‐off phase in which the sideward push‐off occurred. Movement variables were obtained from the inertial signals, which were synchronized with the movement phases observed, during videos for analysis.

For the affected skaters, the gathered cramp characteristics were the total length of the cramp movement (seconds), length of its outward movement and subsequent correction (seconds), corresponding angular velocities (rad/s), angle of the outward movement (degrees), and angular rate of change (angular frequency in rad/s) of the cramp movement.

### Statistical Analysis

Matlab software (R2013b; The MathWorks, Inc., Natick, MA) was used for all data and statistical analysis. We obtained magnitudes of angular velocities from the sensor signals. It should be noted that this method gives directional data in the local foot coordinate system, but that angular velocity magnitudes are independent of that system. Scatterplots were used to access the homogeneity of each subject's gait cycle data regarding their length (seconds). Outliers (>2 interquartile range [IQR] from mean) were removed. Significance thresholds of *P* < 0.05 were used. Q‐Q plots and Shapiro‐Wilk testing were used to test all continuous variables for distribution. In case of normal distribution, the paired‐sample *t* test was used for comparative testing. Otherwise, a nonparametric test (Wilcoxon's signed‐rank test) was applied. For producing averaged gait‐cycle graphs, inequality of individual stride lengths was resolved by creating an equal time axis, using a third‐degree polynomial form of interpolation.

## Results

### Participant Characteristics

The 5 affected subjects (2 male, 3 female) had a median age of 27 (range, 21–51) and 17 (median range: 14–24) years of speed skating experience. After a median period of 14 years (range, 3–22), symptoms of skater's cramp developed. Median disease duration was 4 years (range, 1.5–13.0; Table 1). The nonaffected controls (5 female) were all highly experienced skaters with a median age of 21 (range, 19–37) and a median period of 14 years (range, 12–30) of speed skating experience. The medical history of the affected subjects 2 and 3 included asthma. Subject 5 suffered from tonic‐clonic seizures at age 8; also, she reported migraine and vitamin B12 deficiency. Additionally, she suffered from chronic exertional compartment syndrome on both her legs/feet, for which she had decompressive surgery with subsequent peroneal nerve neuropathy in her left lower leg. Notably, subject 4 reported that his father also suffers from skater's cramp.

**Table 1:** Population characteristics

	Subject 1	Subject 2	Subject 3	Subject 4	Subject 5
Gender	F	F	M	M	F
Age at examination (years)	27	30	51	24	21
Years of ice skating experience	20	16	24	17	14
Main discipline	T	T & Ma	T	T & Ma	T & Ma
Status	Prof	Prof	A	Prof	Prof
Dominant hand/foot	R/L	R/R	R/R	R/R	R/R
Age at onset wobbly foot (years)	23	17	49	16	19
Onset, fully developed symptoms (weeks)	4	1	8	104	0
Complaints manipulable	+	+	+	+	+
Triggers to worsen symptoms	–	+	+	+	+
Triggers to relieve symptoms	+	+	+	+	+

Reported triggers that worsened symptoms were exertion for subjects 2 through 5, stress for subjects 2 and 3, and competition for subjects 2 and 4. Triggers that relieved symptoms were recuperation for subjects 2 through 4, relaxation for subject 1, and using a different ice skating technique for subjects 2, 3, and 5.

F, female; M, male; T, track; Ma, marathon; prof, professional; A, amateur; R, right; L, left.

All affected subjects reported they only suffered during skating. The development of their symptoms was acute with a subsequent chronic course. The symptoms were not progressive after onset in 2 subjects, in 2 it stabilized after 1 to 2 months, and in 1 after 2 years. Two skaters suffered from their right leg, 2 from their left, and 1 reported bilateral problems. In all 5, initial symptoms presented in their nondominant leg. Preceding contributory event(s) were reported in 4 subjects (stress [subjects 2–4], heavy fall [subject 2], and new speed skates [subject 5]). Subjects 2 through 5 reported a subjective change in the sensation of their affected leg after onset, described by all as a feeling of lessened control in the gliding phase. The affected skaters reported that exertion, stress, and performing in competition worsened their symptoms, whereas recuperation and relaxation improved symptoms; 3 subjects also reported that consciously changing their skating technique (“steigerung”) improved their symptoms. All affected subjects had sought medical attention. Sports physiologists, neurologists, sport physicians, physiotherapists, and chiropractors were consulted. Skater's cramp was diagnosed by most parties and no viable therapy was prescribed. For all professional skaters, skater's cramp ended their professional skating careers.

Physical examination of the affected participants showed a subjective numbness on the medial side of the left lower leg in subject 2, minimally reduced position and vibratory sensations in both lower legs in subject 3, and sensory deficits in the left peroneal nerve region in subject 5. Physical examination of the control group was normal.

### Video Analysis

The visual analysis revealed a muscular jerking, that took the form of a patterned repetitive, and rapid exorotation, or endorotation depending on the individual case, followed by a subsequent correction. The jerking movement presented immediately before, during, or after skate placement. Every skater's cramping movement was person specific, with the degree and speed of jerking varying in severity between subjects, while maintaining a very consistent pattern within subjects across gait cycles. For subject 4, we only found aberrative movement on the right leg, with no evidence of bilateral involvement. The jerking movement remained consistent with the addition of extra weight to the skates and also remained visually present during the executing of a different skating technique (“steigerung”). Skaters were never asymptomatic during the skating session.

All affected subjects showed signs of inadequate trunk rotation and translation during the swing phase off the affected leg. Their center of mass (COM) was estimated on the medial side of the affected foot and not, as is expected, on the lateral side of the gliding skate during this phase. The shoulder of the affected body side of subjects 3 and 4 remained in an elevated position throughout their routines. Affected subjects 1 through 4 initiated the arm swing on their affected side earlier, producing a faster and higher swing on their affected side. The controls showed adequate trunk rotation and translation toward the deployed side in the push phase, with COM placed laterally to the gliding skate allowing for the push phase and arm swing to be properly effectuated.

### Movement Variables

Individual values of viable skating cycles obtained from each affected subject's mean total stride length and corresponding swing, glide, and push phase length (seconds) are given in Table 2. Total stride time between legs did not significantly differ for the affected (median, 2.72 [IQR, 0.50]/median, 2.74 [IQR, 0.58] seconds) and control (median, 2.61 [IQR, 0.38]/median, 2.54 [IQR, 0.36] seconds) skaters. However, we did find a significantly longer swing phase for the affected leg compared to the nonaffected leg (median, 1.37 [IQR, 0.35]/median, 1.18 [IQR, 0.24] seconds; *P* < 0.01) and a shorter glide phase (median, 1.09 [IQR, 0.25]/median, 1.26 [IQR, 0.29] seconds; *P* < 0.01). There were no significant differences for these phases in the control group.

**Table 2:** Gait characteristics of the affected and control skaters

		Gait Cycles	Median Stride		Median Swing Phase		Median Glide Phase		Median Push‐Off Phase	
		No.	Length (sec) ± IQR	*P* Value	Length (sec) ± IQR	*P* Value	Length (sec) ± IQR	*P* Value	Length (sec) ± IQR	*P* Value
Affected subject 1	Control leg	16	2.66 ± 0.14	0.86	1.13 ± 0.12	**<0.01** a	1.23 ± 0.06	**<0.01** a	0.30 ± 0.03	**0.03** a
	Affected leg	19	2.66 ± 0.13		1.38 ± 0.06		1.01 ± 0.12		0.27 ± 0.03	
Affected subject 2	Control leg	12	2.89 ± 0.25	0.45	1.33 ± 0.12	0.89	1.32 ± 0.18	0.51	0.24 ± 0.04	0.20
	Affected leg	10	2.98 ± 0.16		1.32 ± 0.08		1.40 ± 0.17		0.26 ± 0.03	
Affected subject 3	Control leg	17	2.39 ± 0.23	0.80	1.01 ± 0.16	0.63	1.09 ± 0.14	0.35	0.28 ± 0.03	**<0.01** a
	Affected leg	16	2.38 ± 0.23		1.03 ± 0.13		1.03 ± 0.12		0.32 ± 0.04	
Affected subject 4	**Affected leg** a	10	2.72 ± 0.53	0.45	1.20 ± 0.42	0.83	1.21 ± 0.27	0.92	0.31 ± 0.04	0.84
	Affected leg	9	2.85 ± 0.56		1.32 ± 0.27		1.22 ± 0.27		0.31 ± 0.03	
Affected subject 5	Control leg	10	3.05 ± 0.15	0.60	1.33 ± 0.07	**<0.01** a	1.44 ± 0.20	**<0.01** a	0.28 ± 0.05	**<0.01** a
	Affected leg	12	3.01 ± 0.18		1.63 ± 0.07		1.15 ± 0.14		0.23 ± 0.02	
Control 1	Left leg	21	2.43 ± 0.21	0.92	1.14 ± 0.19	0.12	1.08 ± 0.17	0.23	0.23 ± 0.05	0.06
	Right leg	21	2.44 ± 0.18		1.10 ± 0.24		1.11 ± 0.27		0.20 ± 0.04	
Control 2	Left leg	22	2.62 ± 0.19	0.93	1.17 ± 0.08	0.13	1.26 ± 0.15	0.31	0.19 ± 0.03	0.58
	Right leg	22	2.61 ± 0.18		1.12 ± 0.11		1.29 ± 0.13		0.20 ± 0.03	
Control 3	Left leg	24	2.28 ± 0.18	0.97	1.01 ± 0.11	0.48	1.07 ± 0.12	0.72	0.20 ± 0.04	0.20
	Right leg	24	2.30 ± 0.16		1.00 ± 0.11		1.07 ± 0.10		0.22 ± 0.05	
Control 4	Left leg	21	2.64 ± 0.21	0.61	1.17 ± 0.11	**<0.01** a	1.22 ± 0.15	0.10	0.26 ± 0.06	0.52
	Right leg	21	2.62 ± 0.19		1.25 ± 0.13		1.14 ± 0.13		0.24 ± 0.04	
Control 5	Left leg	18	2.70 ± 0.21	0.60	1.23 ± 0.13	0.53	1.23 ± 1.22	0.80	0.24 ± 0.03	**0.01** a
	Right leg	22	2.65 ± 0.24		1.27 ± 0.08		1.17 ± 0.19		0.21 ± 0.03	
Affected group	Control leg	65	2.74 IQR 0.58	0.76	1.18 IQR 0.24	**<0.01** a	1.26 IQR 0.29	**<0.01** a	0.28 IQR 0.05	0.52
	Affected leg	66	2.72 IQR 0.50		1.37 IQR 0.35		1.09 IQR 0.25		0.27 IQR 0.07	
Control group	Left leg	106	2.61 IQR 0.38	0.79	1.15 IQR 0.15	0.79	1.19 IQR 0.19	0.73	0.21 IQR 0.04	0.48
	Right leg	110	2.54 IQR 0.36		1.15 IQR 0.23		1.17 IQR 0.23		0.23 IQR 0.06	

Sample size of each subject's studied gait cycles, the median length (seconds) of each stride, and the length of each gait cycle phase. *P* values of <0.05 were considered as statistically significant.

a Subject reports to suffer on both legs.

The push phase did not differ between legs for the affected and control groups.

### Skater's Cramp Characteristics

**Figure 2:**
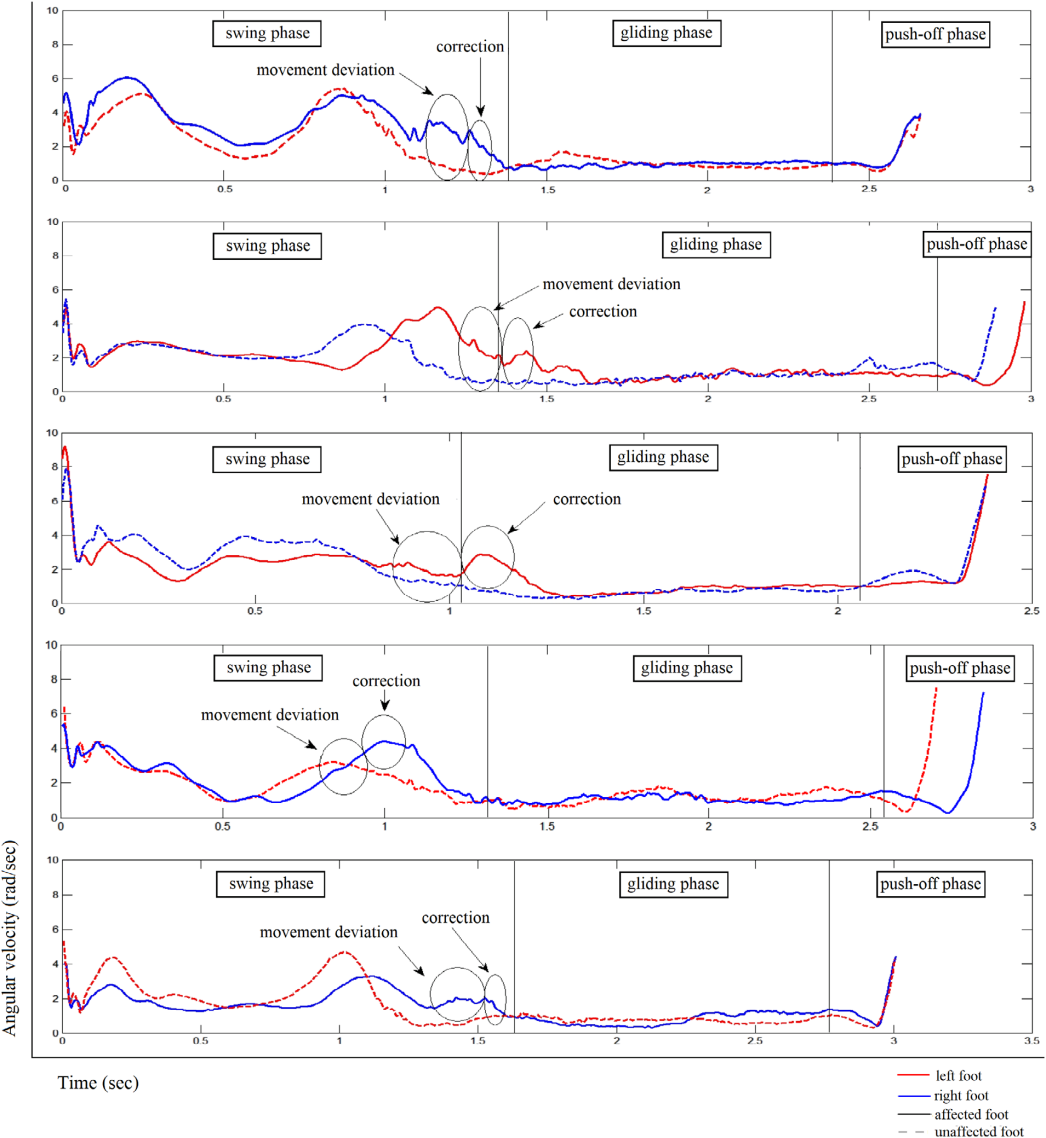
Averaged gait cycle for the angular velocity amplitudes, obtained from the feet sensors from the affected skaters. Distinction between skate‐cycle phases are made. The cramping outward movement and correction are shown. Compared to the nonaffected leg, a significant increase in angular velocity was found during the cramping movement for all subjects (*P* < 0.01). For subject 4, we found no clear aberrative movement in his left leg.

The average skate cycle for angular velocity amplitudes obtained from the foot sensors of all affected subjects is displayed in Figure 2. Two major peaks in angular velocity are observed in all affected subjects during the swing phase, representing the sideward and subsequent forward rotation of the foot during swing phase.

Angular velocity was higher in the affected leg during the cramp deviation (*P* < 0.01), as well as the subsequent correction (*P* < 0.01), for all affected subjects.

Angular frequency of the cramping movement during the routines did not show a significant difference between the nonweighted routine (median, 13.09 [IQR, 6.06] rad/s) and the weighted routine (median, 13.09 [IQR, 5.31] rad/s; *P* = 0.45).

## Discussion

Clinically, our survey reported no generalized complaints; therefore, skater's cramp appears to be task specific. Furthermore, skaters reported their symptoms arose suddenly, after multiple years of skating without previous complaints (3–22 years). Both these important features resemble other forms of TSD, such as musician's dystonia, whose task‐specific symptoms appear abruptly after many years of skilled performance.^4^, ^5^ Another important covariant is stress. Research indicates a strong correlation between TSD and emotional trauma.^6^, ^7^ Three dystonic subjects reported that the period encompassing onset of symptoms was perceived as highly stressful, including an emotionally traumatic sports event preceding onset in 1 subject. Finally, skaters reported proprioceptive sensory changes, including subjective numbness and reduced position/vibratory sensation in their affected leg. Subjective sensory changes are a common symptom of TSD.^8^ Some studies even temporarily elicited dystonic symptoms with vibratory stimuli, suggesting that these sensory changes may not just correlate with TSD, but may be implicated in causing it.^7^

Taken together, task specificity, acute onset, relation to stress, and subjective sensory complaints are positive features for TSD, but can also be explained by functional dystonia.^9^ However, our visual and kinematic analysis indicated that skater's cramp was also an active movement, patterned, and person specific. Combining these visual cues with our clinical observations, in the context of these skilled athletes, makes TSD a more probable explanation than functional dystonia. Furthermore, this feature cluster is observed in other forms of TSD.^10^, ^11^ Nevertheless, identifying new forms of TSD remains a challenge. TSD remains a clinically diagnosed condition, with no systematic quantitative diagnostic criteria as of yet.^12^ For this reason, taken separately, none of our findings are either necessary or sufficient for diagnosis. Taken together, though, they are tentatively indicative or our hypothesis and constitute an important first step in diagnosing skater's cramp, even though more research is required.

Higher angular velocity in the affected leg compared to the unaffected leg seemed to correspond with the visual presentation of an active jerk, although these findings are exploratory and more analysis is needed. One participant reported bilateral involvement, but marked that their symptoms were more serious in one leg, which agreed with our findings, given that we only observed a deviation in the right leg. Although less common, bilateral symptoms can develop in TSD, especially in cases where limbs perform a similar movement.^13^ This notwithstanding, that the jerking visually presented as active (consisting of an extra movement) in all participants is reminiscent of many other forms of TSD, such as musicians for example.^13^ Importantly, this active jerk narrows the scope of our diagnosis, given that it lowers the likelihood that this is a peripheral neuropathy. Peripheral neuropathy would more likely present as a weakening or “giving way” during skate placement, whereas our observations showed the opposite. Subject 5 reported previous peripheral neuropathy and therefore also posed a diagnostic problem. However, here as well, because the condition presented as active, it made the possibility of peripheral neuropathy directly causing the cramp less likely, although it may have factored into its formation, something common in TSD.^13^, ^14^

Skater's cramp was visually observed to be a person‐specific movement pattern, with both active exo‐ and endorotation presenting during skate placement. Also, the amplitude varied greatly with some skaters showing a pronounced jerk, whereas in others it was more subtle. Of note, the visual *movement* of jerking was person specific; the *moment* of symptom onset was highly uniform (during skate placement). In its temporal uniformity and spatial distinctiveness, skater's cramp resembles other forms of TSD, such as in golf. In a recent article on the yips in golf, symptom onset presented uniformly around the moment before hitting the golf ball in putting, whereas the degree of co‐contraction of agonist and antagonist muscles was suggested to be on a spectrum.^11^ Skater's cramp may present on a similar spectrum, almost always happening at the same time, but in a different way for every skater.

Skater's cramp was also patterned, a common aspect of TSD.^10^ The jerk was person specific, but within subjects it presented uniformly across gait cycles, regardless of the intensity or speed. The jerking was so constant, in fact, that visually it seemed to persist consistently throughout the session, irrespective of self‐reports that accelerated skating reduced symptoms. Moreover, a separate research group placed weight on the skater's feet, hypothesizing that it would increase swing amplitude (modeling the limb as a torsion spring^15^). The unexpected outcome revealed that the jerk remained constant in weighted and nonweighted conditions. This is of diagnostic interest to our study, because it makes functional dystonia a less likely explanation. Functional dystonia often fluctuates markedly within one session and is accompanied by varying levels of pain^16^; however, our skaters experienced neither pain nor fluctuating symptoms.

It is hypothesized that the patterned and person‐specific presentation of TSD is attributed to motor engrams that form specifically for the execution of highly practiced tasks.^17^ These engrams are thought to code complex movement sequences in a range of different limb speeds and masses. A recent review implicated the regions coding these engrams in TSD.^12^ Therefore, it is possible that any flawed neural circuitry indicating TSD in areas involved in highly trained motor engrams may present in a range of different speeds and masses, but maintain the same pattern.^17^ We further posit that these movements could likely be person specific, because of the myriad ways these engrams may be corrupted while forming TSD. Further research should focus on the extent to which maladaptive circuitry implicated in motor engrams in highly practiced tasks is indicative of TSD. Clear evidence of the involvement of the central nervous system in skater's cramp may implicate it in this explanation; therefore, some form of brain imaging or neurological test should be considered for future research.

A general visual and kinematic analysis showed that affected skaters suffer from a reduced truncal displacement/body rotation and shorter glide length. Technically, speed skaters may shorten their stride and reduce truncal displacement to reduce the risk of falling when skate placement is unstable. This creates a less‐efficient stride, sacrificing speed. There may be numerous reasons for this. It may be that this defensive stance developed in skaters with existing skater's cramp, as a way to cope with the newly formed unstable skate placement. Alternatively, this asymmetry may have exacerbated skater's cramp. Regardless of which, because these technical problems can, and often do, develop because of any lack of skate stability, it is difficult to gauge their importance in the development of skater's cramp or TSD. One interesting direction for future kinematic research may be ascertaining whether a growth in the severity of asymmetrical problems described above would predict the development of skater's cramp in a larger population.

The clinical characteristics, visual presentation, and movement measurements of skater's cramp makes TSD a possible explanation. This is an important first step in diagnosing a condition that has likely been misdiagnosed and ineffectively treated with invasive surgeries in the past. Future studies of skater's cramp should be focused on using more‐effective diagnostic tools, such as electromyography, to further investigate the hypothesis that TSD may be skater's cramp, as well as possibly leading to treatments for TSD such as botulinum toxin and guided physiotherapy.

## Author Roles

(1) Research Project: A. Conception, B. Organization, C. Execution; (2) Statistical Analysis: A. Design, B. Execution, C. Review and Critique; (3) Manuscript: A. Writing of the First Draft, B. Review and Critique. C. Writing Major Reviews.

B.N.: 1C, 2C, 3A, 3B, 3C

A.H.P.S.: 1A, 1B, 1C, 2A, 2B, 3A, 3C

S.H.: 1A, 1B, 1C, 2B, 3A

R.Z.: 1C, 2C, 3B

E.O.: 1A, 1B, 2C, 3B

M.A.J.T.: 1A, 1B, 1C, 2C, 3C

## Disclosures

### Ethical Compliance Statement

The Medical Ethical Committee of the University Medical Hospital of Groningen (UMCG) reviewed the study and ruled IRB approval was not necessary for this study (M13.147574); therefore, the authors of this piece confirm that approval of an institutional review board was not required for this work. The authors of this piece confirm that written informed consent was obtained from all participating subjects. Furthermore, we confirm that we have read the Journal's position on issues involved in ethical publication and affirm that this work is consistent with those guidelines.

### Funding Sources and Conflicts of Interest

The authors report no sources of funding and no conflicts of interest.

### Financial Disclosures for previous 12 months

MAJT has received grants from the European Fund for Regional Development from the European Union (01492947) and the province of Friesland, ZONMW‐TOP (91218013), Dystonia Medical Research Foundation, from Stichting Wetenschapsfonds Dystonie Vereniging, from Fonds Psychische Gezondheid, from Phelps Stichting, and an unrestricted grant from Actelion.

## Supporting information


**Video S1.** Video example of a Dutch speed skater (subject 2) with skater's cramp affecting her left leg. Notice the abnormal movement just before putting the foot on the ice. Four strides are at normal video speed and subsequently four at half speed.
